# Association between maternal mortality and caesarean section in Ethiopia: a national cross-sectional study

**DOI:** 10.1186/s12884-020-03276-1

**Published:** 2020-10-06

**Authors:** Ayele Geleto, Catherine Chojenta, Tefera Taddele, Deborah Loxton

**Affiliations:** 1 School of Public Health, College of Health and Medical Sciences, Haramaya University, Harar, Ethiopia; 2 Research Centre for Generational Health and Ageing, School of Medicine and Public Health, Faculty of Health and Medicine, the University of Newcastle, Newcastle, Australia; 3 Health System and Reproductive Health Directorate, the Ethiopian Public Health Institute, Addis Ababa, Ethiopia

**Keywords:** Maternal mortality ratio, caesarean section rate, association, Ehiopia

## Abstract

**Background:**

Several studies concluded that there is a reduction of maternal deaths with improved access to caesarean section, while other studies showed the existence of a direct association between the two variables. In Ethiopia, literature about the association between maternal mortality and caesarean section is scarce. This study was aimed to assess the association between maternal mortality ratios and caesarean section rates in hospitals in Ethiopia.

**Methods:**

Analysis was done of a national maternal health dataset of 293 hospitals that accessed from the Ethiopian Public Health Institute. Hospital specific characteristics, maternal mortality ratios and caesarean section rates were described. Pearson’s correlation coefficient was used to determine the direction of association between maternal mortality ratios and caesarean section rate, taking regions into consideration. Presence of a linear association between these variables was declared statistically significant at p-value < 0.05.

**Results:**

The overall maternal mortality ratio in Ethiopian hospitals was 149 (95% CI: 136–162) per 100,000 livebirths. There was significant regional variation in maternal mortality ratios, ranging from 74 (95% CI: 51–104) per 100,000 livebirths in Tigray region to 548 (95% CI: 251-1,037) in Afar region. The average annual caesarean section rate in hospitals was 20.3% (95% CI: 20.2–20.5). The highest caesarean section rate of 38.5% (95% CI: 38.1–38.9) was observed in Addis Ababa, while the lowest rate of 5.7% (95% CI: 5.2–6.2) occurred in Somali region. At national level, a statistically non-significant inverse association was observed between maternal mortality ratios and caesarean section rates. Similarly, unlike in other regions, there were inverse associations between maternal mortality ratios and caesarean section rates in Addis Ababa, Afar Oromia and Somali, although associations were not statistically significant.

**Conclusions:**

At national level, a statistically non-significant inverse association was observed between maternal mortality ratios and caesarean section rates in hospitals, although there were regional variations. Additional studies with a stronger design should be conducted to assess the association between population-based maternal mortality ratios and caesarean section rates.

## Background

Caesarean section (CS) is a surgical procedure used when women experience complications during pregnancy and childbirth in order to save the lives of mothers and babies. CS may be a helpful strategy in meeting Sustainable Development Goal 3 (SGD 3) to reduce the maternal mortality ratio (MMR) below 70 per 100.000 livebirths [1]. Because an estimated 15% of all pregnancies end in obstetric complications, at least these complicated births need to occur in health facilities, which can provide basic and comprehensive emergency obstetric and neonatal care (EmONC), including CS [2, 3]. In low-income countries, where surgical capacity is inadequate, improved access to emergency CS is associated with decreased maternal deaths [3].

In sub-Saharan African (SSA) countries, where maternal health care systems are weak and not well designed, prevalence of obstetric complications and associated maternal deaths remain substantially high [4]. Although the World Health Organization (WHO) previously recommended a population based CS rate between 5–15%, the recent WHO Statement on CS rates identified that CS is associated with short and long-term risks both for mothers and newborns, especially in settings where there is a shortage of capacity to conduct safe surgery. Thus, CS should be undertaken for all women in medically indicated need rather than striving to achieve specific rates. Only maternity care providers can identify women ‘in need’ of CS [2, 5].

There are two extreme situations in the continuum of maternal health care: too little, too late (TLTL) and too much, too soon (TMTS) [6]. TLTL is a situation where there are inadequate resources to provide standard care, which results in high maternal mortality. TMTS includes the unnecessary overuse of non-evidence-based interventions. As facility births increase, so does the recognition that TMTS causes harm, as this increases health costs, disrespects and abuses to the beneficiaries [3]. Therefore, as high rates of CS are considered TMTS, a higher rate of CS with no appropriate medical indication is not associated with a lower rate of maternal mortality [7].

Several studies of the association between maternal mortality ratio (MMR) and mode of birth have yielded inconsistent findings [8, 9]. In a study from Latin America, higher maternal deaths were associated with higher rates of CS as compared to vaginal birth with odds ratios ranging from 1.6 to 7.08 [10]. Similarly, a Brazilian study indicated that births by CS were associated with a three-fold higher in maternal mortality as compared to vaginal delivery [7]. A report of the WHO global survey conducted in nine Asian countries also showed that higher rates of CS were associated with higher MMR [9]. A similar finding was reported in the Netherlands, a high-income country [11]. The higher rate of maternal deaths after CS as compared to vaginal delivery may be attributed to postpartum haemorrhage, complications of anaesthesia and postoperative sepsis [12, 13]. However, all of these studies are cross sectional and unable to conclude the observed associations were causal.

In countries with limited access to comprehensive emergency obstetric care, a significant reduction in MMR was observed through improved access to emergency CS [14, 15]. For example, in India, CS rates increased from 17.4% in 2010 to 22.7% in 2013, and this has been associated with a significant reduction in maternal mortality in that period [15]. A systematic review of CS in SSA showed maternal haemorrhage after CS, (pre-)eclampsia, complications of anaesthesia, sepsis and embolism were associated with higher fatality rates after CS than after vaginal birth [16].

Most scholars in the field of maternal health have reached a consensus that higher CS rates are associated with lower maternal mortality, until a specific threshold, above which the association between the two variables may be reversed [17]. This consensus needs to be verified by analysing representative large datasets, as most literature assessed this correlation by using small sample sizes. In Ethiopia, there is limited literature on the association between MMRs and CS rates. In the country, similar studies were not conducted according to the authors’ knowledge. Therefore, this study aimed to assess the association between MMRs and CS rates in all hospitals in Ethiopia in order to generate evidence for policy and practice for maternal health improvement. This is crucial for Ethiopia, where population-based MMR was 412/100,000 livebirths in 2016 [18] and hospital-based MMR was 350/100,000 livebirths in 2014 [19].

## Methods

### Study Area and period

This study was designed to analyse secondary data of the Ethiopian EmONC assessment survey of 2016. Data about all maternal and neonatal health services in hospitals from 1st January 2015 to 31st December 2015 were retrospectively collected from May to December 2016. The Ethiopian Public Health Institute (EPHI) conducted this survey to assess the status of maternal and neonatal health indicators in Ethiopia [20]. Ethiopia is a multicultural country located in the horn of Africa. The country is a Federal Democratic Republic of nine regional states, namely, Tigray, Afar, Amhara, Oromia, Somali, Benishangul-Gumuz, Southern Nations Nationalities and People Region (SNNPR), Gambella, and Harari and two city administrations (Addis Ababa city administration and Dire Dawa city council). With a population of 109,302,118, Ethiopia is the second most populous country in Africa after Nigeria.

The Ethiopian health system is structured into a three-tier healthcare system, which includes primary, secondary and tertiary level health care. These levels work together through a referral network. Primary level health care comprise of primary health care units (PHCU) that are networked with district hospitals. A PHCU consists of one health center (HC) and five satellite health posts (HPs). A HC, serving approximately 25,000 people, is used as referral center for HPs, the most proximal health facility to the community and serving approximately 5,000 people. HCs are also serving as practical training sites for health extension workers (HEWs), who are health care providers working in HPs. Primary district hospitals provide inpatient and ambulatory services, including comprehensive emergency obstetric care for a population of up to 100,000. Secondary level health care consists of a general hospital and provides general health services for an average of 1,000,000 people. It serves as referral center for primary level healthcare and training center for health officers, nurses and emergency surgeons. Tertiary level health care is the highest level and has a specialized hospital, which serves an average of five million people. It provides specialized services and is used as a referral center for general hospitals [21]. CS is provided in hospitals at all levels, in primary hospitals by emergency surgical officers (health officers trained in emergency surgery) [22] in consultation with obstetricians and in general and tertiary hospitals by obstetricians. Cases are referred to higher levels of care based on severity.

In the last two decades, significant maternal and child health improvements have been observed as the government of Ethiopia invested mainly in health system strengthening. As a result, in 2016, the MMR reduced to 412 per 100,000 live births from a very high baseline of 1,400 per 100,000 live births in 2000 (a 69% reduction). Total fertility rate dropped from 7.7 to 1990 to 4.1 in 2014, which may be explained by an increased contraceptive prevalence rate from 3–42% in the same period. In Ethiopia, trends of maternal health utilization have been increasing over the last two decades. For example, antenatal care from skilled providers increased by 46% over the last 14 years (from 28% in 2005 to 74% in 2019). Over the same period, births in health facilities increased by 43% from 5% in 2005 to 48% in 2019, and the percentage of women who meet the Safe Motherhood Program’s recommendation of receiving postnatal care checks within two days of birth increased to 34% in 2019 from 4.6% in 2005 [23, 24].

### Study participants

Generally, all public and private health facilities, which offered EmONC services, were included in the survey. All 293 private and public hospitals in Ethiopia were among the 3,804 facilities in the survey. This analysis used data of the 293 hospitals’ maternal health indicators, such as number of obstetric complications, maternal deaths, total births, live births and number of CS. The majority of the hospitals were located in the four largest regional states: Oromia (24.9%), SNNPR (20.5%), Amhara (19.1%) and Tigray (13.3%). Slightly more than half, 160 (54.6%), were primary district hospitals, while specialized tertiary hospitals accounted for 30 (10.2%) (Table 1).

**Table 1:** Regional distribution of hospitals in the 2016 Ethiopian EmONC survey

Regions	Type of hospitals			
	**Primary**	**General**	**Specialized**	**Total**
Addis Ababa	3	21	9	33
Afar	5	1	0	6
Amhara	41	10	5	56
Benishangul-Gumuz	1	2	0	3
Dire Dawa	2	3	1	6
Gambella	0	1	0	1
Harari	0	5	1	6
Oromia	41	25	7	73
SNNP	43	13	4	60
Somali	4	6	0	10
Tigray	20	16	3	39
**Nationally**	**160**	**103**	**30**	**293**

### Inclusion criteria

All maternal deaths and all births by CS in the 293 hospitals in 2015 were included in the analysis, irrespective of the type, managing authority and location of the hospitals. Hospitals were included in the survey according to the following eligibility criteria: (1) the hospital provided delivery services in the last 12 months; and (2) the hospital was functional at the time of the data collection period.

### Nature of the data

EPHI collected data about performance of the EmONC signal functions in all Ethiopian hospitals using a standardized questionnaire. Data from hospitals’ registers and patient records included number and mode of births, number of women admitted with specific obstetric complications, and number of all maternal deaths. The dependent variable is MMR in hospitals. The association between CS rates and magnitude of MMR was calculated. Assessment of the association between the dependent and the independent variables was performed by taking regional variation into account.

Healthcare professionals with at least a bachelor degree were recruited and deployed to conduct data collection. During recruitment, factors such as prior experience with data collection, clinical experience and level of education were considered. They were given intensive training on interview techniques, survey tools, field procedures and a detailed review of the questionnaire. The survey was conducted under close supervision of the Technical Working Group (TWG) consisting of Averting Maternal Death and Disability (AMDD), Ethiopian Ministry of Health (MOH) and other collaborators including UNICEF, JHPIEGO, JSI, and the Ethiopian Midwives Association (EMA). In addition, regional coordinators supervised the data collection process and conducted spot-checking to ensure data quality.

### Operational definitions

- **Direct causes of obstetric deaths** are those resulting from obstetric complications of pregnancy, childbirth and puerperium, from interventions, omissions, incorrect treatment, or from a chain of events resulting from any of the above. In this study, the following were considered as major direct causes of obstetric deaths: Antepartum Haemorrhage (APH), Postpartum Haemorrhage (PPH), retained placenta, prolonged/obstructed labour, ruptured uterus, severe eclampsia/preeclampsia, postpartum sepsis, complications of abortion and ectopic pregnancy.
- **Indirect obstetric deaths** are those resulting from previously existing diseases or diseases that developed during pregnancy and which were not due to direct obstetric causes, but aggravated by the physiological effects of pregnancy [25]. These conditions include malaria, anaemia, hepatitis, HIV/AIDS, and other nonspecific potentially life-threatening conditions.

#### Data analysis

Initially, descriptive analysis, including frequency tables, percentages and ratios, was conducted to present facility specific characteristics, MMRs and CS rates. Then, Pearson’s correlation coefficient was performed to assess linear association between MMRs and CS rates, considering regions as covariate. Data analysis was performed with Stata version 15 software. The direction of a linear association between variables was measured using Pearson’s correlation coefficient and an association was declared statistically significant at *p*-value < 0.05.

## Results

### Obstetric complications and maternal deaths

The 2016 national survey identified that out of all 335,054 total births in all 293 hospitals, 78,195 (23.3%) women were admitted with all types of obstetric complications. Most cases (68,002; 87.0%) were admitted with direct obstetric complications. In 2015, 481 maternal deaths occurred in all Ethiopian hospitals; 435 were due to direct causes, while 46 were due to indirect causes. The top three direct causes of maternal deaths were hypertensive disorders of pregnancy (27.8%), postpartum haemorrhage (18.6%) and ruptured uterus (9.4%) (Table 2).

**Table 2:** Obstetric complications and maternal deaths in Ethiopian hospitals in 2015

Obstetric complications	Women affected		Causes of Maternal Deaths	
	**Number**	Percent*	**Number**	**Percent**^**a**^
**Direct (obstetric)**				
Antepartum Haemorrhage (APH)	4,462	6.6	23	5.3
Postpartum Haemorrhage (PPH)	2,811	4.1	81	18.6
Retained placenta	2,201	3.2	4	0.9
Prolonged/obstructed labour	15,875	23.3	25	5.8
Ruptured uterus	1,516	2.2	41	9.4
Postpartum sepsis	1,460	2.2	17	3.9
Severe preeclampsia/eclampsia	7,912	11.6	121	27.8
Abortion complications	2,042	3.0	6	1.4
Ectopic pregnancy	1,801	2.7	0	0.0
Other^b^	27,922	41.1	117	26.9
**Total**	**68,002**	**100.0**	**435**	**100.0**
**Indirect (non-obstetric)**				
Malaria	165	1.6	1	2.2
Anaemia	1,260	12.4	19	41.3
Hepatitis	100	1.0	4	8.7
HIV/AIDS	6,249	61.3	4	8,7
Other indirect causes^c^	2,419	23.7	18	39.1
**Total**	**10,193**	**100.0**	**46**	**100.0**

^a^Percentages for columns were calculated for direct and indirect causes of death separately

^b^Other direct causes include other obstetric complications such as complications of anaesthetic, thromboembolism, and other management related complications; and the underlying causes of direct obstetric complications including premature rupture of membrane and breech presentation, which necessitate hospitals admission and might finally end in direct obstetric complications such as sepsis and obstructed labour

^c^These include nonspecific potentially life-threatening indirect obstetric conditions, including heart disease

### Regional distribution of maternal mortality ratios

The overall MMR in Ethiopian hospitals in 2015 was 149 (95% CI: 136–162) per 100,000 livebirths. There was a significant regional variation ranging from 74 (95% CI: 51–104) per 100,000 livebirths in Tigray region to 548 (95% CI: 251-1,037) per 100,000 livebirths in Afar region **(**Table 3).

**Table 3:** Regional distribution of maternal mortality ratios among hospitals in Ethiopia, 2015

Regions in Ethiopia	Livebirths in hospitals (n)	Maternal deaths in hospital (n)	MMRs per 100,000 livebirths with 95% CI
Addis Ababa	45,049	44	98 (71–131)
Afar	1,643	9	548 (251-1,037)
Amhara	43,899	95	216 (175–265)
Benishangul Gumuz	3,338	4	120 (33–307)
Dire Dawa	5,330	8	150 (65–296)
Gambella	1,667	6	360 (132–782)
Harari	4,823	15	311 (174–513)
Oromia	97,105	142	146 (123–172)
SNNPR	68,422	91	133 (107–163)
Somali	8,181	34	416 (288–580)
Tigray	44,367	33	74 (51–104)
**Total**	**323,824**	**481**	**149 (136–162)**

*MMR* Maternal Mortality Ratio, *SNNPR* Southern Nations Nationalities and People Region

### Regional distribution of caesarean section rates

In Ethiopia, overall, 335,054 births were registered in all hospitals in 2015. The majority (241,512; 72.1%) were spontaneous vaginal deliveries (SVD), while CS accounted for 68,021 (20.3%) of all births and the remaining were assisted vaginal deliveries (AVDs). CS rates ranged from as high as 38.5% (95% CI: 38.1–38.9%) in the capital city Addis Ababa to as low as 5.7% (95% CI: 5.2–6.2%) in Somali region, the most pastoral region of Ethiopia (Table 4).

**Table 4:** Regional distribution of caesarean section rates among hospitals in Ethiopia, 2015

Regions in Ethiopia	Number of women who gave birth in hospitals (n)	Caesarean birth (n)	Caesarean birth Rate (%) (95% CI)^a^
Addis Ababa	46,876	18,037	38.5 (38.1–38.9)
Afar	1,681	129	7.6 (6.4–9.1)
Amhara	46,379	10,317	22.2 (21.8–22.6)
Benishangul Gumuz	3,319	700	21.1 (19.7–22.5)
Dire Dawa	5,507	1,421	25.8 (24.6–26.9)
Gambella	1,729	135	7.8 (6.6–9.2)
Harari	5,399	1,296	24.0 (22.9–25.2)
Oromia	100,398	18,694	18.6 (18.4–18.9)
SNNPR	69,824	11,484	16.4 (16.2–16.7)
Somali	8,545	488	5.7 (5.2–6.2)
Tigray	45,397	5,320	11.7 (11.4–12.0)
Total	**335,054**	**68,021**	**20.3 (20.1–20.4)**

*SNNPR* Southern Nations Nationalities and People Region

^a^CS rate was calculated using number of total births in hospitals as denominator

### Causes of death among women who had caesarean section

**Fig. 1:**
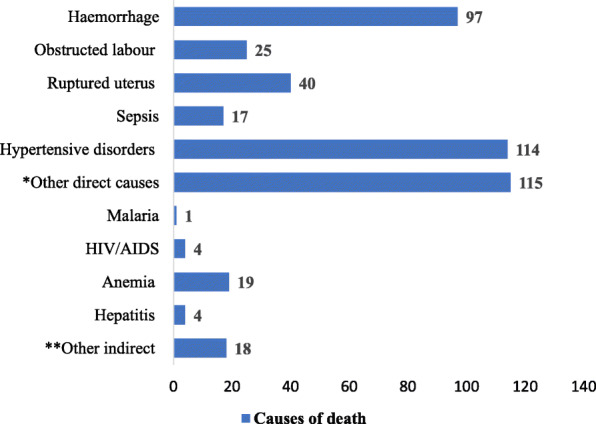
Causes of death among women who had CS in hospitals in Ethiopia in 2015. *Other direct causes: these includes maternal complications such as organ failure, complications of anaesthetic, thromboembolism, and other problems including premature rupture of membrane and breech presentation, which might finally end in direct obstetric complications such as sepsis and obstructed labour. **Other indirect: this incudes other nonspecific potentially life threatening indirect obstetric conditions including heart disease, tuberculosis and incidental causes such as accidents

Four hundred and fifty-four of the 68,021 women who had CS died (case fatality rate 6.7 per 1000 CS). After 267,033 vaginal births, 27 women died (case fatality rate 0.1 per 1000 vaginal births). Risk of death was significantly higher among women who had CS than after vaginal birth [Relative Risk 66.7 (95% CI: 44.6–97.1)]. Death in women who had CS were directly or indirectly related to the procedure of CS. Death due to direct obstetric complications accounted for 408 (89.8%), while the remaining deaths are attributed to indirect causes. Hypertensive disorders of pregnancy and maternal haemorrhage were the two leading direct causes of maternal deaths among women who had CS. While anaemia is the leading indirect cause, hepatitis, HIV/AIDS and other indirect causes of maternal mortality also account for significant numbers of death (Fig. 1).

### Distribution of caesarean section rates and maternal mortality ratios by region

The highest MMR was observed in Afar region followed by Somali and Gambella regions. Conversely, in these regions CS rates were low. In Amhara and Harari regions, both MMRs and CS rates were high while Dire Dawa had high CS rate with moderate MMR. In Addis Ababa, where the highest CS rate was observed, MMR was very low, but Tigray region had the lowest MMR with lower CS rates. While 38.5% of births were conducted by CS in Addis Ababa, in Tigray region CS accounted for only 11.7% of the births although in both regions nearly equal numbers of births occurred in 2015 (Table 5).

**Table 5:** Distribution of caesarean section rates and maternal mortality ratios among hospitals in Ethiopia by regions, 2015

Regions in Ethiopia	Maternal mortality ratios (95% CI)	Caesarean section rates (95% CI)
Addis Ababa	98 (71–131)	38.5 (38.1–38.9)
Afar	548 (251-1,037)	7.7 (6.4–9.1)
Amhara	216 (175–265)	22.3 (21.9–22.7)
Benishangul Gumuz	120 (33–307)	21.1 (19.7–22.6)
Dire Dawa	150 (65–296)	25.8 (24.7–26.9)
Gambella	360 (132–782)	7.8 (6.6–9.2)
Harari	311 (174–513)	24.0 (22.9–25.2)
Oromia	146 (123–172)	18.7 (18.4–18.9)
SNNPR	133 (107–163)	16.5 (16.2–16.8)
Somali	416 (288–580)	5.7 (5.2–6.2)
Tigray	74 (51–104)	11.7 (11.4–12.0)
**Total**	**149 (136–162)**	**20.3 (20.2–20.5)**

### Association of MMRs and caesarean section rates

**Fig. 2:**
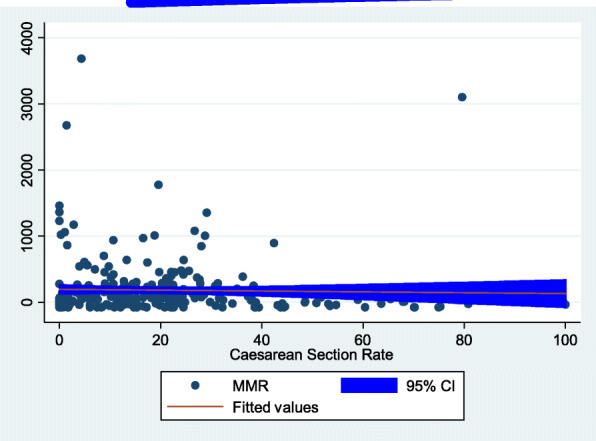
The correlation of maternal mortality ratio and caesarean section rate in Ethiopia, 2015

Correlation analysis was performed between MMRs and rates of CS in all hospitals during 2015. At national level, an inverse association was observed between MMRs and CS rates in hospitals although the association was very weak and statistically not significant (r = -0.03, *p* = 0.52) (Fig. 2). Similarly, there were inverse associations between MMRs and CS rates in Addis Ababa, Afar, Oromia and Somali regions (negative Pearson’s correlation coefficient), but again not statistically significant (*p* > 0.05). Nevertheless, there was a statistically significant and direct association between MMRs and CS rates in Amhara (r = 0.43, *p* = 0.003) and Tigray (r = 0.47, *p* = 0.002) regions. Direct association between the two variables was also detected in Benishangul Gumuz, Dire Dawa, Harari, and SNNP regions (positive Pearson’s correlation coefficient), but none of the associations were statistically significant (*p* > 0.05) (Table 6).

**Table 6:** The correlation coefficient for maternal mortality ratios and caesarean section rates among Ethiopian regions, 2015

MMRs in Ethiopian regions	CS rates in Ethiopian regions											
	**AA**	**Afar**	**Amhara**	**BG**	**DD**	**Gambella**	**Harari**	**Oromia**	**SNNP**	**Somali**	**Tigray**	**Total**
AA	-0.25											
Afar		-0.43										
Amhara			0.43 *									
BG				0.93								
DD					0.11							
Gambella						- ^a^						
Harari							0.19					
Oromia								-0.08				
SNNP									0.17			
Somali										-0.03		
Tigray											0.47 *	
Total												-0.03

The correlation coefficient for MMRs and CS rates for each region were worked out by using the magnitude of both parameters of the regions. Overall correlation coefficient was calculated using the magnitude of the national MMR and CS rate and the numbers represent Pearson’s correlation coefficient (r)

*AA* Addis Ababa, *BG* Benishangil Gumuz, *DD* Dire Dawa, *SNNP* Southern Nations, Nationalities and People, *MMR* Maternal Mortality Ratio, *CS* Caesarean Section

* Pearson’s correlation coefficient *p* < 0.01

^a^ Observations are insufficient to generate correlation coefficient

## Discussion

In 2015, the overall national MMR in all Ethiopian hospitals was 149/100,000 livebirths. Our findings showed a substantially lower MMRs as compared to the Ethiopian Demographic and Health Survey in 2016 [18]. Although there are no similar studies in the country, current findings are also lower than that found in a small scale study in a specialized [19], general [26] and rural (for directly admitted women) hospital [27] in Ethiopia. Our estimate is much lower compared to findings of a systematic review of hospital-based studies in other sub-Saharan Africa, where the pooled MMR was 957 per 100,000 livebirths [28]. It is also much lower than the findings of a Nigerian study where MMR was 2085/100,000 livebirths among eight included hospitals from different regions [29], but still much higher than in high-income countries [30]. Relatively lower MMRs in hospitals may reflect the effectiveness of several maternal health interventions such as Maternal Death Review and Reporting system (MDSR), which is being implemented in Ethiopia. MDSR is a health reform used to continuously notify, review, analyse and respond to maternal deaths in order to take action to prevent similar deaths in the future [31].

There was a huge regional variation in the magnitude of MMRs. This is comparable with results of the Ethiopian Demographic and Health Survey [18]. Regional variations in MMR were also reported in a Nigerian study and a systematic review in sub-Saharan African countries [28, 29]. An explanation could be huge disparity in access to healthcare among different regions of Ethiopia. Shortage of clinical skills and unavailability of a strong referral system in some regions could also play a role. Differences in the type of hospitals available in different regions could also account for regional variations [28].

Prevalence of obstetric complications among women admitted in hospitals was very high (23.4%) and surprisingly higher than the 15% WHO estimate [32]. Higher prevalence of obstetric complications compared to previous findings could be a consequence of a referral system whereby complicated maternity cases are referred to hospitals. Consistent with an Eritrean study, hypertensive disorders of pregnancy were the leading cause of maternal mortality [33]. Similarly, this study revealed that postpartum haemorrhage, prolonged labour and postpartum sepsis were the predominant causes of maternal mortality, supported by several small-scale studies in Ethiopia [19, 34] and findings of a systematic review of literature [35].

The current study revealed a nationwide hospital-based CS rate of 20.3%. Although high, this is much lower than a CS rate of 47.6% in Dessie referral hospital, northern Ethiopia [36]. It is also lower than a CS rate of 27.6% in Attat Hospital in southern Ethiopia, [37]. The rate of CS in Addis Ababa city administration is much higher than the finding of a previous study, where a CS rate of 19.2% was reported [38], although the most recent study reported a comparable CS rate (38.3%) in the city [39]. Our study included data from all hospitals, while previous studies used smaller samples and were conducted only in urban centers.

Huge regional variations in CS rates exist in Ethiopia. Somali, Afar and Ganbella regions, where CS rates were substantially low and access to maternal health services inadequate [18]. In contrast, CS rates were considerably high in Addis Ababa, Harari and Dire Dawa, which are the urban centers of the country. Our findings were comparable with previous study reports, where Addis Ababa, Harar and Dire Dawa had the highest CS rates and Somali region the lowest [40]. In line with our findings, several previous hospital-based studies revealed that CS rates were very high in these urban centers [38, 40, 41]. These regional disparities may be attributed to better access to surgical services with overuse of the procedure and higher prevalence of elective CS in large cities of the country [6].

Type of hospital and available skills to provide CS might explain regional disparities. The majority of hospitals in pastoral regions including Somali and Afar are primary district hospitals, where emergency surgical officers perform CS. In contrast, in major regions such as Addis Ababa, Oromia, Tigray and Amhara, there are several referral teaching hospitals where highly skilled professionals, including obstetricians, perform CS. In these major regions, there is a strong referral linkage among different hospitals because there are many primary, general and referral hospitals as opposed to the pastoral regions. Furthermore, the number of hospitals available in some regions are inadequate to serve the regions’ population. For example, Gambella region has only one hospital.

A weak and statistically non-significant inverse linear association was observed between MMR and CS rate at national level, although there were significant regional disparities. Our findings in these regions are supported by a study in Turkey, where an inverse association was observed between maternal mortality and CS rates [42]. This may be explained by the fact that most often CS is performed in response to emergency medical complications that saved lives of most women [43].

Nevertheless, the association between MMR and CS rate was unevenly distributed among the regional states of Ethiopia. The current study revealed that there was a direct association between MMRs and CS rates in Amhara, Benishangul Gumuz, and SNNP regions. Although Tigray region had the lowest MMR and low CS rate, there was statistically significant direct association between the two variables in this region. This may indicates that CS is not the solely option to reduce MMR. Similarly, CS rates were high both in Dire Dawa city administration and in Harari region while MMR was relatively lower in Dire Dawa than Harari region. Although the association between MMRs and CS rates in these regions was direct, this association was statistically not significant. Available evidence indicated that higher rates of CS are associated with lower MMR only when the CS rate is below 10% [17]. Therefore, observing higher MMRs in these regions was not surprising because similar to the current findings, previous studies also indicated CS rates in these regions considerably exceeded 10% [36, 41, 44].

Findings of a previous systematic review conducted in Latin America is also consistent with our findings [10]. Esteves-Pereira AP et al. 2016 reported a three-fold increased risk of maternal mortality with caesarean compared to vaginal birth in Brazil [7]. In low-income countries, higher risks of death with higher CS rates may be attributed to complications with anaesthesia, lack of surgical skills and postoperative sepsis[12, 13]. Women in low-income countries may not get effective prophylactic antibiotics and rigorous skin preparation before CS [45]. This increases the probability of postoperative infection and contributes to higher maternal mortality after CS [13, 45].

In the current study, the case fatality rate for women who undergone CS was very high as compared to vaginal births. This is comparable with findings of a systematic review and meta-analysis conducted in low- and middle-income countries where the risk of maternal death in women who had CS was 7.6 per 1000 women (10.9 per 1000 women in sub-Saharan Africa) [46]. Although the risk of maternal death is usually higher after CS than after vaginal birth both in low [46] and high income [11, 47] countries, the magnitude was greater in our study than in most previous studies. This may be because this study included only hospitals with data where complicated cases are managed while vaginal births can be provided at lower level facilities. Most mothers who experienced obstetric emergencies are referred to hospitals from lower level facilities and arrive in hospitals with advanced conditions. The majority of these women become candidates for CS, indicating higher risk of death after CS than after vaginal births. This evidence indicates a need for further in-depth review of maternal deaths after CS in order to identify where the chain of events started that led to death [11].

### Strengths and limitations of the study

This study has several strengths. We used national representative data of all hospitals in Ethiopia. Therefore, generalization can be applied to all regions of Ethiopia. These findings may also be useful for other low-income countries with similar demographic and socio-economic characteristics. From the commencement of the survey, experts from national and international partners were involved in data collection and management processes, which enhanced data quality. However, this study suffered from the usual limitation of a cross-sectional study in that causal associations could not be concluded. Since we calculated hospital-based CS rates based on the WHO Statement on Caesarean Section Rates, the current study did not estimate population level CS rates; hence, we are unable to generalize our findings to the general population. Although vaginal births can be attended in health centres that cannot provide CS, women who experience emergency obstetric complications are often referred to hospitals. This is a possible explanation for the high prevalence of obstetric complications and low numbers of maternal deaths after vaginal births. Maternal deaths in this study represent only the numbers, which were registered in the hospitals’ logbooks. Therefore, we were challenged while interpreting our findings due to shortage of similar literature, since the majority of previous studies were population.

## Conclusions

This study demonstrated that at national level in Ethiopia there was a statistically non-significant inverse association between CS rates and MMRs in hospitals. There were significant disparities in MMRs among regions in Ethiopia. The observed inverse associations between MMRs and CS rates in some regions suggest that access to emergency CS is an important intervention to reduce maternal deaths. In addition, direct associations between MMRs and CS rates in major regions indicate excessive CS-rates may not be helpful when performed in the absence of maternal indications. Further studies with a strong design should be conducted to investigate the causal relationship between maternal deaths and CS rates. Further in-depth review of maternal deaths after CS is required in order to identify where the chain of events started that lead to death after CS.

## Data Availability

The data that support the findings of this study are available from EPHI, but restrictions apply to the availability of these data, which were used under license for the current study, and so are not publicly available. Data are however available from the authors upon reasonable request and with permission of the EPHI.

## Abbreviations

AIDS: Acquired Immune Deficiency Syndrome
APH: Antepartum Haemorrhage
AMDD: Averting Maternal Death and Disability
CS: Caesarean Section
EMA: Ethiopian Midwives Association
EmOC: Emergency Obstetric Care
EmONC: Emergency Obstetric and Newborn Care
EPHI: Ethiopian Public Health Institute
HC: Health Centre
HEW: Health Extension Worker
HIV: Human Immunodeficiency Virus
HPs: Health Post
JHPIEGO: Johns Hopkins Program for International Education in Gynaecology and Obstetrics
JSI: John Snow Inc
MMR: Maternal Mortality Ratio
MOH: Ministry of Health
PHCU: Primary Health Care Unit
PPH: Postpartum haemorrhage
SDGs: Sustainable Development Goals
SNNPR: Southern Nations, Nationalities and People Region
SVD: Spontaneous Vaginal Delivery
TLTL: Too Little Too Late
TMTS: Too Much Too Soon
TWG: Technical Working Group
WHO: World Health Organization
UNICEF: United Nation’s Children’s Fund
